# The impact of allergic contact dermatitis on the inflammatory response and repair in wound healing process

**DOI:** 10.3389/fmed.2025.1524198

**Published:** 2025-02-19

**Authors:** Wei Zhang, Jiabao Xu, Songyan Qu, Hui Peng

**Affiliations:** ^1^Department of Dermatology, Henan Provincial People's Hospital, Zhengzhou University People's Hospital, Henan University People's Hospital, Zhengzhou, Henan, China; ^2^Physical Education College of Zhengzhou University, Zhengzhou, Henan, China; ^3^Department of Dermatology, Shaanzhou District People's Hospital, Sanmenxia, China; ^4^Department of Dermatology, The Second Affiliated Hospital of Zhengzhou University, Zhengzhou, Henan, China

**Keywords:** allergic contact dermatitis, inflammatory response, cellular response, wound healing process, inflammatory markers

## Abstract

**Background:**

Skin trauma and the subsequent wound healing process present significant challenges for healthcare systems and patients globally. Allergic contact dermatitis (ACD) was a delayed-type hypersensitivity reaction that can disrupt the normal wound repair process due to prolonged inflammation and immune dysregulation. However, the specific impact of ACD on the inflammatory response and repair in wound healing remains incompletely understood. This study aimed to investigate the influence of ACD on the inflammatory response and repair during the wound healing process.

**Methods:**

This study was a retrospective cohort study. A total of 120 patients with skin trauma treated at Henan Provincial People’s Hospital from January 2023 to December 2023 were included. There were 69 cases of control and 51 cases of ACD. Inclusion and exclusion criteria were defined, and various indicators, including patient data, inflammatory factors, cell detection, and wound healing assessment, were measured and analyzed using appropriate statistical methods.

**Results:**

The study revealed significant differences between the control and ACD groups. ACD was associated with higher levels of TNF-*α*, IL-6, IL-1β, C-reactive protein, and IL-8 compared to control (*p* < 0.05). Additionally, ACD group exhibited increased counts of macrophages, neutrophils, T lymphocytes, B lymphocytes, and mast cells compared to the control group (*p* < 0.05). Moreover, ACD was linked to delayed wound closure time and differences in the distribution of healing degrees (*p* < 0.05). Correlation analysis indicated significant associations among ACD, inflammatory markers, cellular responses, wound closure time, and healing degree (*p* < 0.05).

**Conclusion:**

The study demonstrates that ACD exerts a substantial impact on the inflammatory response, cellular components, and wound healing parameters in the context of skin trauma. The heightened levels of inflammatory markers, altered cellular responses, and delayed wound closure observed in ACD patients underscore the need for targeted interventions tailored to optimize wound repair in this population.

## 1 Introduction

Skin trauma and its subsequent repair process represent a significant burden on healthcare systems and patients worldwide (1, 2). However, the presence of certain dermatologic conditions, such as allergic contact dermatitis (ACD), can significantly impact the normal wound repair process (3). ACD was a delayed-type hypersensitivity reaction (DTH, after contact with a certain substance, skin redness, swelling, induration, etc. appeared over a period of time) involving immune system dysregulation in response to specific allergens or irritants, resulting in inflammatory skin reactions (4–6).

The inflammatory phase of wound healing was a critical stage characterized by the recruitment of immune cells, the release of pro-inflammatory cytokines (PIC, A cytokine that triggers an inflammatory response and enhances the immune system’s response), and the modulation of the local tissue microenvironment to orchestrate the clearance of debris and the initiation of the repair process (2, 7, 8). However, in the presence of ACD, the dysregulated immune response may lead to an exaggerated and protracted inflammatory phase, potentially disrupting the coordinated progression of wound repair (9, 10). This prolonged inflammation can impede the transition to the proliferative and remodeling phases of wound healing, ultimately contributing to delayed or impaired tissue repair (11). The altered immune reactivity in ACD may impact the function of resident immune cells, fibroblasts, and other stromal cells, further complicating the repair process (1, 12, 13). The recruitment, activation, and function of immune cells, such as neutrophils, macrophages, and lymphocytes, play pivotal roles in modulating the local tissue environment and regulating the progression of wound healing (14–17). Coco-Viloin M et al. (18) studied the healing of surgical incisions in a 26-year-old patient with hidradenitis suppurativa who underwent autologous skin grafting, and the results showed that the patient’s long-term exposure to panthenol in cosmetics produced ACD, leading to a significant delay in the healing of surgical incisions. Lopez DV et al. (19) explored the role of IL-22 in skin diseases and found that IL-22 promoted the proliferation of skin keratinocytes and dermal fibroblasts and played an important role in wound healing and preventing skin infection. ACD resulted in decreased IL-22 secretion. Inhibition of IL-22-induced anti-apoptotic genes (such as Bcl-2 and Bcl-xl) and matrix metalloproteinases (such as MMP1/3) enhances cell proliferation, thereby inhibiting remodeling of the epidermis and dermis.

Therefore, this study aimed to investigate the impact of ACD on the inflammatory response and repair in the context of wound healing, with a specific focus on exploring alterations in inflammatory markers, cellular responses, and wound repair parameters.

## 2 Materials and methods

### 2.1 Study population

This study was a retrospective cohort study. A total of 120 patients with skin trauma treated at our hospital from January 2023 to December 2023 were randomly sampled, who had complete data from wound formation to healing at our hospital. Among them, there were 69 cases of control group (no-ACD) and 51 cases of ACD group.

### 2.2 Inclusion and exclusion criteria

#### 2.2.1 Inclusion criteria

Meets the definition of traumatic wounds, including physical trauma (burns, scalds, mechanical injuries, etc.), chemical trauma (chemical burns, extravasation injuries from medication, etc.); Age ≥ 18 years; Judged to be wounds amenable to debridement and potential healing after comprehensive assessment; Wound area between 1 to 20 cm^2^, depth ≥ 0.50 cm; Patients with normal mental and cognitive function; Complete medical records.

#### 2.2.2 Exclusion criteria

Wounds with secondary osteomyelitis or malignant lesions; Wounds with active bleeding; Presence of systemic comorbidities affecting wound healing such as autoimmune diseases, malnutrition, advanced tumors, multi-organ failure, poorly controlled diabetes, etc.; Individuals receiving treatment with steroids, immunosuppressants, anti-tumor agents, or other therapies affecting wound healing; Patients who have participated in other clinical trials in the three months prior to the current study.

### 2.3 Diagnosis of ACD

ACD can be diagnosed if the following criteria were met: rashes commonly occur at the site of contact; the morphology of the rash often varies depending on the nature of the contact substance, with sensitizers usually presenting with well-defined borders and predominantly as erythema, swelling, papules, blisters and even bullosa, while irritants often cause erythema, vesicles or bullae, erosion, and even necrosis; presence of itching and burning sensation, and in severe cases, pain, fever, and other systemic symptoms; a self-limiting course, with rashes caused by certain sensitizers subsiding within 1 to 2 weeks after removal of the causative agent; and positive results in patch tests for the sensitizer. The diagnosis of ACD was: Symptom observation, patch test (by covering the patient’s skin with cotton or linen impregnated with the allergen to check for allergens), trigger test (using a small amount of allergens to simulate a natural reaction to see if the patient has the same allergic reaction), skin test (by injecting or applying a specific substance to the skin to see if there was an allergy to the substance). Patch test was an important way to diagnose contact dermatitis. The site of the inflammatory response was exposed to substances that were non-irritating. Treatment focused on finding the cause of the allergy and avoiding re-contact of certain parts of the body with the substance. The diagnosis of ACD was made by independent examination of two physicians.

### 2.4 Testing indicators

#### 2.4.1 Patient data

General patient data were systematically retrieved from the medical records, including age, gender, BMI, smoking history, alcohol consumption history, comorbidities, wound type, wound location, wound depth, wound area, blood tests, and wound healing status. All patients with wounds in this study developed ACD after the injury. In all patients, ACD spread to the vicinity of the wound. Our research team closely monitored the occurrence of ACD in the hospital nursing department, and all patients enrolled in the study were screened and enrolled and had their blood taken within 3 days of the occurrence of ACD.

#### 2.4.2 Inflammatory factors

Blood samples were taken for inflammation and cell detection on the day after all patients signed the informed consent form. All patients fasted for 8 h, and the next morning, 5 mL of venous blood was collected from the elbow and centrifuged at room temperature at 3,000 *r*/min for 5 min. Enzyme-linked immunosorbent assay was used to determine the levels of inflammatory factors in the two groups, including tumor necrosis factor-*α* [TNF-α (ab181421, Abcam, USA)], interleukin-4 [IL-4 (ab215089, Abcam, USA)], interleukin-6 [IL-6 (ab178013, Abcam, USA)], interleukin-8 [IL-8 (ab214030, Abcam, USA)], interleukin-10 [IL-10 (ab185986, Abcam, USA)], interleukin-1β [IL-1β (ab214025, Abcam, USA)], and the levels of C-reactive protein [CRP (ab260058, Abcam, USA)] using immunoturbidimetry.

#### 2.4.3 Cell detection

Three milliliters of fasting venous blood were collected from the patients’ elbow and subjected to serum separation. The neutrophil count was determined using a fully automated hematology analyzer (XE2100, SYSMEX, Japan). Lymphocytes were marked with fluorescent antibodies and analyzed using a flow cytometer (FC500, Beckman, USA) (20). T lymphocytes were marked with CD3 and CD4, while B lymphocytes were marked with CD19. Mast cells (MC) and macrophages were stained using the routine ABC method (21). Positive cells exhibited brownish-yellow granules in the cytoplasm and/or nucleus. Five random tissue fields (including interstitium and tissue) were selected at high magnification for cell counting, and their mean was calculated.

#### 2.4.4 Wound healing assessment

The observation and recording of wound healing time and degree were conducted for both groups of patients. The wounds of patients were assessed by three researchers on a daily basis to reduce the risk of bias. The evaluation criteria for wound healing status were as follows: optimal – complete wound healing with no significant color difference from the surrounding skin; good – basic wound healing with slight scar pigmentation; fair – basic wound healing with evident scar pigmentation; poor – no wound healing. The wound healing time was the time from the formation of the wound to the occurrence of all other healing states except for the condition of “poor - no wound healing.” We kept a close eye on the wound by taking pictures and taking various measurements every day. There was no secondary wound healing in all patients included in this study.

### 2.5 Statistical methods

The data analysis was conducted using SPSS 29.0 statistical software (SPSS Inc., Chicago, IL, USA). Categorical data were expressed in the form of [*n* (%)]. For sample sizes ≥40 and theoretical frequency T ≥ 5, the chi-square test was applied using the basic formula. When the sample size was ≥40 but the theoretical frequency was 1 ≤ T<5, the chi-square test was performed using the corrected formula. For sample sizes <40 or theoretical frequency T < 1, statistical analysis was performed using Fisher’s exact probability method. The normality of continuous variables was assessed using the Shapiro–Wilk test. For normally distributed continuous variables, they were represented in the form of (X ± s), and the corrected variance *t*-test was utilized. Non-normally distributed data were represented in the form of median (25th percentile, 75th percentile), and the Wilcoxon rank-sum test was applied. Both sides of *p* < 0.05 were considered to indicate statistical significance. The relationship between continuous variables such as TNF-*α*, IL-6, IL-1β, C-reactive protein, IL-8, wound closure time, and the impact of inflammatory response and repair during wound healing process was analyzed using Pearson correlation. The relationship between categorical variables such as healing degree and the impact of inflammatory response and repair during the wound healing process was analyzed using Spearman correlation.

## 3 Results

### 3.1 General data and demographic characteristics

In this study, the independent sample *T*-test was used to calculate the Power efficacy value, and the results showed that based on the premise that the class I error was 0.05, the class II error was 0.19, and the Power value was 0.9, in order to achieve the difference of the above studies with scientific judgment, the sample size of group 1 and Group 2 should both reach or exceed 46, as shown in Table 1. The demographic characteristics and general data of the two patient groups were summarized in Table 2 A total of 69 patients were included in the control group, with a mean age of 38.52 years (± 5.24) and a gender distribution of 31 males (44.93%) and 38 females (55.07%). In the ACD group, consisting of 51 patients, the mean age was 39.81 years (± 4.75), and the gender distribution was 26 males (50.98%) and 25 females (49.02%). The two groups showed comparable BMI, smoking history, drinking history, comorbidities, wound type, wound location, wound depth, and wound area, with no statistically significant differences observed (*p* > 0.05). These findings suggest that the demographic and general data were well-balanced between the two groups, laying the foundation for comparative assessment of the impact of ACD on the inflammatory response and repair in the wound healing process.

**Table 1:** Power analysis results.

Alpha value (Type I error)	Beta value (Type II error)	Power value (1-Beta value)	Group 1 Sample Size (n1)	Group 2 Sample Size (n2)	Sample size ratio (n1/n2)
0.05	0.19	0.900	46.000	46.000	1

**Table 2:** General data and demographic characteristics of the two patient groups.

Parameter	No allergic contact dermatitis (*n* = 69)	Allergic contact dermatitis (*n* = 51)	t/χ^2^	*p*
Age (years)	38.52 ± 5.24	39.81 ± 4.75	1.409	0.162
Gender (M/F)	31 (44.93%)/38 (55.07%)	26 (50.98%)/25 (49.02%)	0.222	0.637
BMI (kg/m^2^)	23.38 ± 2.14	23.86 ± 1.92	1.297	0.197
Smoking history	19 (27.54%)	13 (25.49%)	0.002	0.967
Drinking history	10 (14.49%)	9 (17.65%)	0.046	0.830
Comorbidities (%)	7 (10.14%)	7 (13.73%)	0.100	0.752
Hypertension	11 (15.94%)	10 (19.61%)	0.078	0.780
Diabetes	10 (14.49%)	6 (11.76%)	0.027	0.871
Hyperlipidemia	38.52 ± 5.24	39.81 ± 4.75	1.409	0.162
Wound type				
Surgical	28 (40.58%)	23 (45.10%)	None	0.969
Traumatic	21 (30.43%)	16 (31.37%)		
Pressure ulcer	7 (10.14%)	4 (7.84%)		
Burn	7 (10.14%)	5 (9.80%)		
Other	6 (8.70%)	3 (5.88%)		
Wound location				
Face and neck	7 (10.14%)	6 (11.76%)	1.217	0.875
Lower abdomen	10 (14.49%)	5 (9.80%)		
Upper abdomen	10 (14.49%)	10 (19.61%)		
Limbs	35 (50.72%)	26 (50.98%)		
Other	7 (10.14%)	4 (7.84%)		
Wound depth				
Subcutaneous fat layer	28 (40.58%)	19 (37.25%)	0.200	0.905
Muscle layer	31 (44.93%)	25 (49.02%)		
Cartilage layer	10 (14.49%)	7 (13.73%)		
Wound area				
<3 cm^2^	28 (40.58%)	18 (35.29%)	0.471	0.790
3–10 cm^2^	14 (20.29%)	10 (19.61%)		
>10 cm^2^	27 (39.13%)	23 (45.10%)		

### 3.2 Inflammatory markers

In the comparison of inflammatory markers between the control group and ACD groups, statistically significant differences were observed in the levels of TNF-*α* (15.85 ± 4.21 pg./mL vs. 17.89 ± 4.56 pg./mL, *t* = 2.503, *p* = 0.014), IL-6 (10.16 ± 2.34 pg./mL vs. 11.32 ± 3.45 pg./mL, *t* = 2.069, *p* = 0.042), IL-1β (7.96 ± 1.87 pg./mL vs. 8.76 ± 2.12 pg./mL, *t* = 2.154, *p* = 0.034), C-reactive Protein (4.56 ± 1.21 mg/L vs. 5.21 ± 1.89 mg/L, *t* = 2.167, *p* = 0.033), and IL-8 (12.13 ± 2.65 pg./mL vs. 13.55 ± 4.21 pg./mL, *t* = 2.124, *p* = 0.037) (Table 3). However, no statistically significant differences were found in the levels of IL-4 (11.27 ± 3.45 pg./mL vs. 11.54 ± 2.98 pg./mL, *t* = 0.459, *p* = 0.647) and IL-10 (14.32 ± 4.21 pg./mL vs. 13.87 ± 3.11 pg./mL, *t* = 0.683, *p* = 0.496) between the two groups. These results indicate that ACD was associated with altered levels of specific inflammatory markers, suggesting the impact on the inflammatory response in the wound healing process.

**Table 3:** Inflammatory markers in control group vs. ACD group.

Parameter	No allergic contact dermatitis (*n* = 69)	Allergic contact dermatitis (*n* = 51)	*t*	*p*-value
TNF-α (pg/mL)	15.85 ± 4.21	17.89 ± 4.56	2.503	0.014
IL-4 (pg/mL)	11.27 ± 3.45	11.54 ± 2.98	0.459	0.647
IL-6 (pg/mL)	10.16 ± 2.34	11.32 ± 3.45	2.069	0.042
IL-1β (pg/mL)	7.96 ± 1.87	8.76 ± 2.12	2.154	0.034
IL-10 (pg/mL)	14.32 ± 4.21	13.87 ± 3.11	0.683	0.496
C-reactive Protein (mg/L)	4.56 ± 1.21	5.21 ± 1.89	2.167	0.033
IL-8 (pg/mL)	12.13 ± 2.65	13.55 ± 4.21	2.124	0.037

### 3.3 Cellular response

In comparing the cellular response between the control group and ACD groups, statistically significant differences were observed in the counts of macrophages (141.35 ± 20.83 cells/mm^2^ vs. 152.46 ± 25.15 cells/mm^2^, *t* = 2.57, *p* = 0.012), neutrophils (83.14 ± 15.42 cells/mm^2^ vs. 90.15 ± 18.73 cells/mm^2^, *t* = 2.182, *p* = 0.032), T lymphocytes (185.36 ± 30.16 cells/mm^2^ vs. 202.34 ± 35.42 cells/mm^2^, *t* = 2.761, *p* = 0.007), B lymphocytes (58.27 ± 12.79 cells/mm^2^ vs. 63.58 ± 14.56 cells/mm^2^, *t* = 2.08, *p* = 0.04), and mast cells (25.16 ± 10.45 cells/mm^2^ vs. 30.45 ± 12.36 cells/mm^2^, *t* = 2.473, *p* = 0.015) (Table 4). These findings suggest that ACD may have a significant impact on the cellular components of the inflammatory response in the wound healing process, as reflected by alterations in the counts of specific immune cells.

**Table 4:** Cellular response in control group vs. ACD group.

Parameter	No allergic contact dermatitis (*n* = 69)	Allergic contact dermatitis (*n* = 51)	*t*	*p*
Macrophage count (cells/mm^2^)	141.35 ± 20.83	152.46 ± 25.15	2.57	0.012
Neutrophil count (cells/mm^2^)	83.14 ± 15.42	90.15 ± 18.73	2.182	0.032
T Lymphocyte count (cells/mm^2^)	185.36 ± 30.16	202.34 ± 35.42	2.761	0.007
B Lymphocyte count (cells/mm^2^)	58.27 ± 12.79	63.58 ± 14.56	2.08	0.04
Mast cell count (cells/mm^2^)	25.16 ± 10.45	30.45 ± 12.36	2.473	0.015

### 3.4 Wound healing

In comparing wound healing parameters between the control group and ACD groups, notable differences were observed (Table 5). The mean wound closure time was 9.75 ± 2.32 days in the control group, while it was 10.63 ± 2.14 days in the ACD group (*t* = 2.151, *p* = 0.034), indicating a delayed wound closure in the latter group. Additionally, the distribution of healing degrees differed significantly between the two groups (χ^2^ = 7.351, *p* = 0.038), with a higher proportion of patients in the control group classified as having excellent healing (59.42% vs. 45.10%) and a lower proportion classified as having fair (5.80% vs. 21.57%) or poor healing (1.45% vs. 3.92%) compared to the ACD group. These results suggest that ACD may have an impact on the wound healing process, leading to a prolonged wound closure time and differences in the distribution of healing degrees.

**Table 5:** Wound healing in control group and ACD groups.

Wound healing parameter	No allergic contact dermatitis (*n* = 69)	Allergic contact dermatitis (*n* = 51)	t/χ^2^	*p*
Wound closure time (days)	9.75 ± 2.32	10.63 ± 2.14	2.151	0.034
Healing degree				
Excellent	41 (59.42%)	23 (45.10%)	None	0.038
Good	23 (33.33%)	15 (29.41%)		
Fair	4 (5.80%)	11 (21.57%)		
Poor	1 (1.45%)	2 (3.92%)		

### 3.5 Correlation analysis

The correlation analysis revealed several significant associations between inflammatory response, cellular response, repair status, and ACD in the studied population (Table 6). Specifically, TNF-*α* levels exhibited a positive correlation with ACD (*r* = 0.227, R^2^ = 0.052, *p* = 0.013), as did IL-6 (*r* = 0.197, R^2^ = 0.039, *p* = 0.031), IL-1β (*r* = 0.198, R^2^ = 0.039, *p* = 0.03), C-reactive Protein (*r* = 0.208, R^2^ = 0.043, *p* = 0.023), IL-8 (*r* = 0.204, R^2^ = 0.042, *p* = 0.025), macrophage count (*r* = 0.236, R^2^ = 0.056, *p* = 0.009), neutrophil count (*r* = 0.203, R^2^ = 0.041, *p* = 0.027), T lymphocyte count (*r* = 0.252, R^2^ = 0.064, *p* = 0.005), B lymphocyte count (*r* = 0.192, R^2^ = 0.037, *p* = 0.036), mast cell count (*r* = 0.227, R^2^ = 0.052, *p* = 0.013), wound closure time (*r* = 0.192, R^2^ = 0.037, *p* = 0.036), and healing degree (*r* = 0.218, R^2^ = 0.048, *p* = 0.017). These findings underscore the intricate interplay between ACD, inflammatory and cellular responses, and the status of wound repair.

**Table 6:** Correlation analysis of the inflammatory response, cellular response, repair status, and ACD.

Parameter	*r*	R^2^	*p*
TNF-α (pg/mL)	0.227	0.052	0.013
IL-6 (pg/mL)	0.197	0.039	0.031
IL-1β (pg/mL)	0.198	0.039	0.03
C-reactive Protein (mg/L)	0.208	0.043	0.023
IL-8 (pg/mL)	0.204	0.042	0.025
Macrophage count (cells/mm^2^)	0.236	0.056	0.009
Neutrophil count (cells/mm^2^)	0.203	0.041	0.027
T Lymphocyte count (cells/mm^2^)	0.252	0.064	0.005
B Lymphocyte count (cells/mm^2^)	0.192	0.037	0.036
Mast cell count (cells/mm^2^)	0.227	0.052	0.013
Wound closure time (days)	0.192	0.037	0.036
Healing degree	0.218	0.048	0.017

## 4 Discussion

The results of the present retrospective cohort study provide valuable insights into the impact of ACD on the inflammatory response and repair in the wound healing process. The study population of 120 patients with skin trauma, including 69 cases of control and 51 cases of ACD, allowed for a comprehensive assessment of the differences in inflammatory markers, cellular responses, and wound healing outcomes between the two groups. The findings shed light on the intricate interplay between ACD, inflammatory responses, cellular components, and wound repair.

In our study, we observed notable differences in the levels of inflammatory markers between the control and ACD groups. Specifically, ACD was associated with higher levels of TNF-*α*, IL-6, IL-1β, C-reactive protein, and IL-8 when compared to control. These findings suggest that ACD may contribute to an altered inflammatory milieu at the site of skin trauma, leading to increased levels of PIC and acute phase reactants. This heightened inflammatory response in ACD has important implications for the modulation of the wound healing process, as excessive inflammation can potentially impair the orderly progression of tissue repair and regeneration (22). These results are consistent with those of other recent expert studies. Gendrisch F et al. (23) studied regulators of skin aging and inflammation, It was found that lutetin can inhibit skin aging and promote wound healing by inhibiting pro-inflammatory factors IL-1β, IL-6, IL-8, IL-17, IL-22, TNF-*α*, COX-2 and regulating NF-κB, JAK–STAT, TLR signaling pathways. Ayuob N et al. (24) studied the therapeutic effect of pumpkin fruit extract (PE) on contact dermatitis (CD) in depressed rats. The results showed that PE had anti-inflammatory effects by significantly down-regulating pro-inflammatory cytokines (TNF-α, IL-6, COX-2, iNOS) and significantly up-regulating antioxidants (SOD, GPX and CAT) (*p* < 0.001), and promoted wound healing with CD.

Importantly, our study also revealed significant differences in the cellular response to skin trauma between the control and ACD groups. We observed increased counts of macrophages, neutrophils, T lymphocytes, B lymphocytes, and mast cells in the ACD group compared to the control group. This suggests that ACD may elicit a distinct cellular immune response at the site of skin trauma, involving a broader spectrum of immune cells. The recruitment and activation of these immune cells in the context of ACD may influence the local tissue microenvironment and contribute to the protracted inflammatory phase of wound healing.

Furthermore, our study demonstrated a delayed wound closure time and differences in the distribution of healing degrees in patients with ACD compared to those with control. These findings highlight the potential impact of ACD on the overall wound repair process. Prolonged wound closure time and differences in healing degrees may signify a compromised or aberrant repair response in the presence of ACD. The delayed resolution of skin trauma in the context of ACD underscores the need for a comprehensive understanding of the underlying pathophysiological mechanisms to optimize the management of skin trauma in these patients. These results are consistent with earlier and recent studies of ACD. Fanning JE et al. (25) conducted a meta-analysis on the healing of surgical incisions in allergic contact dermatitis, and the results showed that most surgical wounds would have a significant response to contacts, and ACD could easily occur at the incision, which was very unfavorable to the healing and recovery of the incision. Blanchard G et al. (26) carried out molecular biological tests on ACD mouse wound animal model. The results showed that cutaneous keratinocytes can up-regulate the junctional adhesion molecule-like protein (JAML) ligand coxsackievirus and adenovirus receptor (CXADR) after exposure to the allergen. This makes it difficult for the wound to heal.

The correlation analysis revealed intricate interplay among ACD, inflammatory and cellular responses, and wound repair status. Several significant associations were identified, emphasizing the complex network of interactions influencing the dynamics of the wound healing process in the context of ACD. Positive correlations between inflammatory markers, cellular response elements, wound closure time, and healing degree underscore the multifactorial nature of the impact of ACD on wound repair.

ACD was a DTH reaction involving T cell activation and pro-inflammatory cytokine release, leading to prolonged inflammation at the site of the allergic reaction, which can disrupt the normal process of wound healing (27). This prolonged inflammation can impede the normal progression of the wound healing process, leading to delayed or impaired tissue repair (28). Additionally, the presence of ACD can alter the local skin microenvironment, affecting the function of resident immune cells, fibroblasts, and other cells involved in wound healing. Individuals with ACD may also have heightened immune responses to certain antigens, further exacerbating the inflammatory response in the context of skin trauma (29). This altered immune reactivity can impact the coordinated cellular and molecular events necessary for effective wound repair (30). The complex interplay between immune dysregulation, prolonged inflammation, and altered tissue microenvironment disrupts the normal cascade of events involved in wound healing (31).

The study has several strengths, including its robust sample size, comprehensive assessment of inflammatory markers and cellular responses, and its focus on real-world clinical scenarios. However, it was important to acknowledge certain limitations. As a retrospective cohort study, causal relationships cannot be inferred from the observed associations. Additionally, the study’s findings were limited to the specific cohort and may not be generalizable to broader populations. The retrospective design also limits causal inference, selection bias that can result from unspecified sampling techniques, and results that rely on subjective assessments of wound healing (e.g., “excellent” to “poor”), which tend to bias results. Future prospective studies with larger and diverse patient cohorts were warranted to validate and extend the current findings. Targeted therapies to reduce inflammatory markers in ACD patients will be investigated, and prospective larger studies will be conducted to validate the results, and the genetic or molecular mechanisms of the observed healing delay will be explored.

## 5 Conclusion

In conclusion, our study provides relatively valuable insights into the impact of ACD on the inflammatory response and repair in the wound healing process. The distinct alterations in inflammatory markers, cellular responses, and wound healing parameters observed in the context of ACD clarified the need for a multifaceted approach to the management of skin trauma in these patients. Further research focusing on delineating the underlying mechanisms and exploring targeted interventions to optimize wound repair in the presence of ACD. Ultimately, a deeper understanding of the complex interplay between ACD and the wound healing process could advancing personalized the effective management strategies for skin trauma.

## Data Availability

The original contributions presented in the study are included in the article/supplementary material, further inquiries can be directed to the corresponding author.
